# Prevelance of upper extremity lymphedema and risk factors in patients with mastectomy: Single-center, observational, cross-sectional study

**DOI:** 10.4274/tjod.galenos.2020.33734

**Published:** 2020-10-02

**Authors:** Tuba Tülay Koca, Gökmen Aktaş, Mehmet Emre Kurtgil

**Affiliations:** 1 Sütçü İmam University Faculty of Medicine, Department of Physical Medicine and Rehabilitation, Kahramanmaraş, Turkey; 2 Sütçü İmam University Faculty of Medicine, Department of Clinic Oncology, Kahramanmaraş, Turkey

**Keywords:** Breast cancer, lymphedema, rehabilitation

## Abstract

**Objective:**

Upper extremity complaints are frequently encountered in breast cancer. It was aimed to investigate the pain, extremity pain, and limitation of motion, lymphedema prevalence, severity, risk factors and quality of life in patients with breast cancer followed by mastectomy in our center.

**Materials and Methods:**

The study included 67 patients with mastectomy. The presence of lymphedema, lymphedema duration, and grade of lymphedema were recorded. Grip strength was measured on both hands using a dynamometer; arm, shoulder and hand problems were evaluated using the disabilities of the arm, shoulder, and hand. Quality of life was assessed using the World Health Organization Quality of Life scale-short form.

**Results:**

The presence of lymphedema was 23.9%; the most common was international society of lymphology grade 1 (76.1%); the median lymphedema duration was 12 (range, 3-72) months. Radical/modified radical mastectomy (58.2%) was the most common type of surgery. Median pain score in the affected extremity according to the visual analogue scale was 2 (minimum: 0/maximum: 7); the presence of shoulder pain was 40.3%; shoulder movement limitation was 7.5%.

**Conclusion:**

It was found that lymphedema had a negative effect on quality of life by affecting shoulder, arm, and hand functions even in the early stages. The recognition of risk factors and signs of upper extremity complications in breast cancer survivors will contribute to rehabilitation success.

**PRECIS:** Upper extremity lymphedema in patients with mastectomy.

## Introduction

Lymphedema is a localized tissue swelling caused by the excessive retention of lymphatic fluid in the interstitial space and is caused by impaired lymphatic drainage. It is classified as primary or secondary. Primary lymphedema is caused by developmental lymphatic vascular anomalies; secondary lymphedema is acquired and is caused by an underlying cause, such as systemic disease, trauma, or surgery. This progressive chronic disease has serious effects on the quality of life of the affected person. It mimics other conditions that cause extremity swelling and is often misdiagnosed. There is no definitive cure for lymphedema. However, with proper diagnosis and management, progression and possible complications can be prevented^(1,2)^.

In the near future, developing countries will face a rapid increase in the number of individuals in the elderly population. In the next 20 years, the average age will reach 50 years and individuals aged over 65 years will form a significant part of society. The incidence of breast cancer increases with age, the risk of breast cancer reaches 0.44% at age 30 years, 3.82% at age 70 years, and 10% at the age of 80 years^(3)^. Recent studies have noted that a large portion of older patients do not receive conventional treatment for breast cancer. They are frequently treated with breast conservation, omitting axillary dissection, radiation therapy, and chemotherapy^(4)^. The development of upper extremity lymphedema following axillary lymph node (LN) dissection in patients with breast cancer is reported in 16-40% of cases. Lymphedema may develop in 3.5% of cases following sentinel LN biopsy alone^(5)^. Regional LN dissection is an important risk factor for the development of upper extremity lymphedema in patients with breast cancer, but there are insufficient tools to accurately measure the risk of lymphedema in individuals. Body mass index (BMI), the extent of axillary surgery, the number of LNs, and the width of the nodal radiation can be counted among the affecting risk factors^(6,7)^. Lymphedema adversely affects the quality of life in young women of active working age who are treated for breast cancer^(8)^.

The prevalence of lymphedema in patients with surgical breast cancer is high and difficult to treat. There is limited literature information about additional risk factors such as LN dissection and BMI, infection, and radiation in its formation^(1,2,3,4,5,6,7,8)^. The aim of this study was to investigate the pain, extremity pain and limitation of motion, lymphedema prevalence, severity, risk factors, and the effect on quality of life in patients with breast cancer followed by mastectomy in our center.

## Materials and Methods

The study was planned prospectively and as cross-sectional reasearch. The study included 67 patients with mastectomy who were followed up in the oncology outpatient clinic. Participants’ age, BMI, smoking, education level, breast cancer diagnosis time, histopathologic type, tumor stage, surgical procedure, postoperative time, number of positive LNs, affected arm, dominant hand, upper extremity diameter difference, presence of lymphedema, lymphedema duration, grade of lymphedema, history of infection in the affected extremity, shoulder pain on the affected side, severity of pain [visual analogue scale, (VAS) 0-10], and presence of shoulder movement limitation were recorded. The presence of lymphedema was determined objectively according to the diameter difference in extremity circumference measurements, which were defined as standard in both arms. Patients without lymphedema were accepted as grade 0. Grip strength was measured in both hands using a dynamometer (kg); arm, shoulder, and hand problems in the affected extremity were evaluated using the disabilities of the arm, shoulder, and hand (DASH) and Tampa scale for kinesiophobia (TSK). Quality of life was assessed using the World Health Organization Quality of Life scale-short form (WHOQOL-bref). Neuropathic symptoms such as dysesthesia and anesthesia were not questioned.

The inclusion criteria were: (1) having undergone mastectomy over the age of 18 years for unilateral breast cancer. The exclusion criteria were (1) a history of cognitive dysfunction, (2) upper extremity orthopedic surgery or trauma, (3) bilateral involvement, and (4) a history of neuropathic or myopathic disease that might cause muscle weakness. Data were recorded by the same experienced physician. The study was approved by the local Medical Research Ethics Committee (protocol no: 2019/185). Informed and written consent was obtained from all participants.

### Outcome Measures

### Clinical Stage of Lympedema

The clinical stage of lymphedema was determined according to the International Society of Lymphology (ISL). Patients without apparent symptoms were graded as stage 0 because all patients who underwent axillary LN dissection were considered to have impaired lymph transport.

Stage 0: A latent or subclinical condition in which limb swelling is not yet evident.

Stage I: An early accumulation of fluid that subsides with limb elevation.

Stage II: Tissue swelling that is not reduced by limb elevation alone. Pitting is manifested in earlier stage II, but the limb may or may not pit in later stage II because excess fat and fibrosis supervene.

Stage III: Lymphostatic elephantiasis in which pitting can be absent and trophic skin changes, such as acanthosis, further deposition of fat and fibrosis, and watery overgrowths, have developed.

### Upper Extremity Diameter Measurements

Lymphedema of the upper extremity was evaluated using the circumferential method. The circumferential upper extremity measurements were performed with the arm abducted at 30°, starting at the level of the carpometacarpal joint, every 5 cm proximal to this point along both extremity^(9)^. Interextremity volume difference was defined as edema.

### DASH Questionnaire

DASH is a self-report questionnaire that detects physical function and symptoms in people with musculoskeletal disorders of the upper extremity. DASH has 30 items, and each item is scored on a Likert scale from 1 to 5 where 1 reflects ‘no difficulty’ and 5 ‘severe difficulty.’ Scores are transformed to a 0-100 scale with higher DASH scores indicating greater disability. This instrument assesses physical functions, symptoms, and social functions. The optional four items related to work or sports activities were not used for this study^(10)^. The Turkish validity and reliability study of DASH has been conducted by Duger et al.^(11)^.

### Grip Strength

The grip strength of the upper extremities was measured using a handheld kg (model 5030J1, Sammons Preston Rolyan, Bolingbrook, IL, USA) in the standardized recommended position by American Society of Hand Therapy, with a rest period of 20 seconds; three trials were performed and the mean values were recorded.

### TSK

Kinesiophobia is a term that was introduced by Miller, Kori and Todd in 1990 at the Ninth Annual Scientific Meeting of the American Pain Society, and it describes a situation where “A patient has an excessive, irrational, and debilitating fear of physical movement and activity resulting from a feeling of vulnerability to painful injury or reinjury.” TSK is a 17-item questionnaire used to assess the subjective rating of kinesiophobia or fear of movement. The original questionnaire was developed to “discriminate between non-excessive fear and phobia among patients with chronic musculoskeletal pain.” Several studies have found the scale to be a valid and reliable psychometric measure. As the score increases, the severity of kinesiophobia increases^(12,13)^.

### WHOQOL-BREF

WHOQOL-BREF produces scores for four domains related to quality of life: physical health, psychological, social relationships and environment. It also includes one facet on overall quality of life and general health. WHOQOL-BREF provides a valid and reliable alternative to the assessment of domain profiles using the WHOQOL-100. It is envisaged that the WHOQOL-BREF will be most useful in studies that require a brief assessment of quality of life, for example, in large epidemiologic studies and clinical trials where quality of life is of interest^(14)^.

### Statistical Analyses

Statistical analyses were performed using the Statistical Package for the Social Sciences for Windows version 20.0 software (IBM Corp., Armonk, NY, USA). The variables were investigated using visual (histograms, probability plots) and analytical methods (Kolmogorov-Smirnov test) to determine whether they were normally disturbed. Analysis of the characteristics of patients was performed using descriptive studies. Analysis of variance was used to compare the groups. Spearman test was used for correlation analysis. A multiple linear regression model was used to identify independent predictors of lympedema presence. A p-value 0.05 was considered as statistically significant.

## Results

The study included 67 women with a median age of 50.4±11.2 years median =36 months who had been diagnosed and usergone surgery for breast cancer. The sociodemographic data of the participants are summarized in Table 1. The presence of lymphedema was 23.9%; the most common was ISL grade 1 (76.1%); the median lymphedema duration was 12 (range, 3-72) months. The majority of the participants were primary school graduates (40.3%). Radical/modified radical mastectomy (58.2%) was the most common type of surgery. The mean number of positive LNs was 13.2±11.5. The median tumor stage was 2 (range, 1-4) and 62.6% were invasive ductal carcinoma. The most frequently involved side was the right arm with a rate of 55.2%. The median pain in the affected extremity according to the VAS scale was 2 (minimum: 0/maximum: 7); the rate of shoulder pain was 40.3%; the rate of shoulder movement limitation was 7.5%; and the mean DASH score was 65.1±20.8. The mean general health score was 6.4±1.6; physical health score was 23.2±4.8; psychological health score was 22 (11-29); social health score was 10.5±2.2; environmental health score was 28.9±5.1; and total WHOQOL score was 89.3±12.7. The mean TSK was 40.4±7.7. Only three patients had a history of infection (4.5%) in the affected extremity.

When we divided the groups into 4 groups according to ISL lymphedema grading, the patients’ number distributions were not homogeneous (grade 0, n=51; grade 1, n=7; grade 2, n=8; grade 3, n=1), pain (VAS) in the affected extremity (p=0.01); shoulder pain (p=0.05); limitation of movement in the shoulder (p<0.001); WHOQOL total score (p=0.01); physical health score (p=0.09); psychological health score (p=0.07); social health score (p=0.08); and environmental health score (p=0.01) were significantly different between the groups (Table 2).

Advanced age was positively correlated with the number of positive LNs extracted and DASH score, and was negatively correlated with hand grip strength hand and social health score. The duration of diagnosis was positively correlated with the duration of lymphedema and negatively correlated with the social health score. The VAS score of the affected extremity was positively correlated with lymphedema duration, WHOQOL total score, and DASH, and was negatively correlated with general health, physical, psychological health score, hand grip stregth. Lymphedema duration was positively correlated with VAS score, social and total WHOQOL score, and negatively with hand grip strength. The number of positive LNs extracted was positively correlated with postoperative duration and TSK, and negatively correlated with hand grip strength. Postoperative duration was positively correlated with lymphedema duration and negatively correlated with social health score. The difference in diameter between extremities was negatively correlated with psychological health score.

Hand grip strength was negatively correlated with DASH and TSK, and positively correlated with general health, physical health, social health, environmental health, and total WHOQOL scores. DASH score was positively correlated with TSK, and negatively correlated with general health, physical health, psychological health, social, environmental and total WHOQOL scores. BMI was negatively correlated with general health and psychological health. Lymphedema grade was positively correlated with extremity diameter difference and duration of lymphedema, and negatively correlated with psychological, environmental health, and total WHOQOL scores. Statistically significant correlation results only are summarized in Table 3.

Multiple logistic regression analysis showed that the number of extracted positive LNs (beta=0.575; p=0.10); DASH score (beta=-0.266; p=0.013); total WHOQOL score (beta=3.712; p=0.001); and the duration of breast cancer diagnosis (beta=-2.257; p=0.031) were found to be significant predictors of lymphedema presence.

## Discussion

Breast cancer is one of the most common cancers among women. Upper extremity lymphedema is the riskiest and most frequent complication that occurs following breast cancer surgery (20%), causing irreversible and functional, psychological, and social problems^(15)^. It is proportional to axillary surgery and radiation. Sentinel LN biopsy is the option for elective axillary LN dissection in patients with clinical node-negative early-stage breast cancer. Other risk factors are obesity and infection. Minimizing axillary surgery and radiation reduces the risk. Early physical therapy, weight loss, skin and nail care after surgery are cornerstones of treatment in early-stage lymphedema. Late-stage lymphedema may benefit from plastic surgery^(16)^. Women with breast cancer also report upper extremity symptoms (shoulder pain, limitation of motion in the shoulder, paresthesia, axillary web syndrome, loss of strength) at rates ranging from 10-64%^(17,18)^. In our study, the frequency of shoulder pain was close to half of the patients and limitation of motion in the shoulder was one-sixth. Pain in the affected extremity was median 2 (VAS: 0-10 cm). Pain, shoulder pain, and limitation of motion in the shoulder were significantly high in the presence of lymphedema and in the affected extremities in advanced grades, and was found to be a positive predictor of lymphedema.

As the number of positive LNs increases, we see that postoperative time and kinesiophobia increase and hand grip strength decreases. This can be explained by the fact that the presence of lymphedema is more common in patients with high LN numbers. In determining the presence of lymphedema, the positive LN number, breast cancer diagnosis time, DASH score showing shoulder, arm and hand functions, and quality of life were found to be important determinants. Accordingly, the high number of extracted LNs and long duration of diagnosis increases the risk of developing lymphedema. In addition, upper limb function and quality of life are negatively affected in these individuals as expected. As the degree of lymphedema increases, we see that the presence and severity of pain in the shoulder (VAS), mobility limitation, and negative effects on quality of life subparameters increase. Accordingly, lymphedema negatively affects the affected limb functions. In the early diagnosis of lymphedema, questioning pain and limitation of motion on the involved side can be a guide for early diagnosis. The symptoms persist for a long time, and even those with mild lymphedema may develop moderate or severe lymphedema. Breast cancer-associated lymphedema can be a transient or permanent condition. Early diagnosis of lymphedema and initiation of a home program including appropriate exercises affect quality of life in patients with breast cancer^(19)^. In our study, quality of life scores in patients with breast cancer were negatively affected by the duration between diagnosis and surgery, BMI, diameter difference, extremity pain, shoulder pain, DASH, and hand grip strength.

Mastectomy seems to be multifactorial in the etiology of lymphedema. However, it appears that even certain known risk factors do not provide information on the development of lymphedema. In the study by Penn et al.^(20)^, more LN metastases, weight gain, and extremity diameter difference were observed as risk factors for the development of persistent lymphedema. In our study, the duration of lymphedema, the number of positive LNs, DASH score, and the duration of breast cancer diagnosis were positive predictors of the presence of lymphedema. BMI and diameter difference were not significant. Despite negative sentinel LN biopsies up to 7 years postoperatively, patients present with arm and shoulder symptoms that affect daily life^(21)^. The development of lymphedema can be as short as 3 months and can be seen after years. Therefore, hand, arm and shoulder symptoms should be followed closely and lymphedema should be detected in the early period.

Secondary lymphedema in cancer treatment is characterized by progressive fibroadipous tissue accumulation, increased infection, and malignancy risk. To date, it has been thought to be associated with impaired collateral lymphatic formation after surgical injury. However, chronic inflammation-related fibrosis plays a key role in recent publications. Lymphatic damage is associated with a chronic immune response (T helper cell) that causes fibrosis and lymphatic leakage, decreased lymphatic pumping, and impaired collateral lymphatic formation^(22)^.

Physical therapy modalities such as self-massage, manual lymphatic drainage, therapeutic physical exercises, compression bandage, elastic compression garments, kinesio tape, pneumatic compression, ultrasonic, electrostatic, extracorporeal shock wave therapy, electrical muscle stimulation and laser therapy are used in the treatment of postmastectomy lymphedema. Although recent studies have not shown superiority over one another, combined therapies in advanced stage lymphedema are recommended^(23,24,25,26,27)^. There are serious developments in alternative and new surgical approaches in the management of lymphedema^(28)^. Methods such as physical activity, acupuncture, healing touch, hypnosis, and music therapy, yoga, tai chi, visual reality, and cognitive behavior therapy are also used in cancer pain^(29)^. Obesity appears to be a risk factor for the development of lymphedema after breast cancer and mastectomy, and preoperative measures should be taken^(30,31)^. Therefore, it is important how the percentage of total fat and BMI affect the measurements that determine lymphedema. For this purpose, tissue dielectric constant method is used for accurate detection of lymphedema associated with breast cancer treatment^(32)^. In our study, BMI was not significantly different in patients with lymphedema, but BMI was negatively correlated with psychological and general health scores. Obesity adversely affects general and psychological health.

Various imaging methods can be used in the diagnosis and treatment of lymphedema. Ultrasound is also helpful in revealing extremity differences in the diagnosis of upper extremity lymphedema^(33)^. In the study by Kilmartin et al.^(34)^, low-level laser therapy in patients with lymphedema has been shown to reduce beneficial effects in breast cancer symptoms and emotional stress. Axillary reverse mapping (ARM) and sentinel LN dissection often involve common and associated lymphatic drainage pathways. According to recent studies, the combination of sentinel LN biopsy and ARM is promising to prevent the development of lymphedema after surgery^(35)^.

According to the study the lymphoedema impact and prevelance - international study in Turkey, most of the Turkish patients were recruited from specialist lymphedema services and were found to be women, housewives, and had secondary lymphedema because of cancer treatment. The duration of lymphedema was commonly <5 years and most of them had ISL grade 2 lymphedema. Cellulitis, infection, and wounds were uncommon. The majority of patients received no treatment or advice before. Most of the patients had impaired quality of life and decreased functionality, but psychological support was neglected. Although most had social health security access to lymphedema centers, access seemed difficult because of distance and cost^(36)^. According to the data of our center, our participants consisted of obese, primary school educated, non-smoking women in their 50s. The median breast cancer was stage 2 and the majority had undergone radical/modified radical mastectomy. The time after diagnosis and surgery was similar (median 3 years). All of the histopathologic findings were invasive ductal carcinoma. The majority of the patients were ISL grade 0 or 1 lymphedema. The Infection rate was low in the affected extremity. A reduction in hand grip strength was observed in patients with both early and advanced grade lymphedema.

Upper extremity lymphedema affects work and sometimes careers. Workplace adaptations can be useful^(37)^. Similar to our study, in the study by Chachaj et al.^(38)^ factors such as upper extremity pain (shoulder and arm), pain in the operated breast, difficulty in arm movements, dermatolymphangitis and a history of chemotherapy were found to be associated with high DASH and low-quality life scores. Lymphedema severity, young age, BMI, and lymphedema localization were not associated with poor outcomes. As expected, the severity of lymphedema was positively correlated with diameter difference and lymphedema duration, and was negatively correlated with quality of life scores. Accordingly, high severity of lymphedema negatively affects the quality of life.

Zou et al.^(39)^ found that lymphedema might occur at the earliest 1 month after surgery and this incidence has increased over time, especially observed in the first year in their study. In our study, the mean duration of lymphedema after surgery was 36 months (the earliest 3 months, the latest 240 months). In the same study, axillary LN dissection, radiotherapy, modified radical mastectomy, positive number of axillary LNs and BMI were found to be independent risk factors for the development of lymphedema. Giray and Akyüz^(40)^ showed that shoulder instability caused caregiver burden and decreased quality of life.

It is very important in the management for early breast cancer by selecting the most suitable surgery mode for every individual patient to cure their disease and to satisfy the patient psychologically. Conservation should be preferred prior to reconstruction whenever possible. The choice of breast conserving surgery and radical mastectomy/modified radical mastectomy in patients with breast cancer is determined by the decision of the physician or patient. In our study, the radical mastectomy/modified radical mastectomy ratio is 58.2% and is compatible with the general literature^(41,42)^. Several factors explain why some women do not develop lymphedema after axillary LN dissection. The reduced lymphatic flow mechanism by the lymphatics alone cannot explain the late onset and selected protected areas (such as hands). Quantitative lymphoscintigraphy indicates that the drainage of the lymphatic flow in the subcutis (where edema is most common) is slower and that the subfascial muscle compartment has a higher lymph flow than the subcutis. Lymphatic congestion lymphoscintigraphy showed the association of edema with decreased contractility in arm lymphatics. Swelling increases as the active lymphatic pump weakens^(43)^.

Kinesiophobia is an irrational fear that is linked to the belief in susceptibility to injury. It is associated with lower physical activity levels. Kinesiophobia adversely affects the compliance of older patients to rehabilitation programs^(44)^. According to our study, kinesiophobia was not significantly different between the groups; TSK was positively correlated with hand grip strength and the number of positive LNs. As seen, kinesophobia is found high in patients whose shoulder, arm and hand functions are more affected. These patients may also develop kinesiophobia to protect the affected extremities from trauma.

### Study Limitations

In this study, chemotherapy or radiotherapy protocols were not taken into consideration. Data originates from a single center, which restricts generalization. In some patients, some questionnaires could not be completed due to low education levels. The majority of our study group consisted of patients with tumor stage 0 or 1. The limited number of patients with advanced stage lymphedema is another limitation of the study.

## Conclusion

In summary, lymphedema is a chronic, progressive condition caused by imbalance in lymphatic flow. Secondary lymphedema is common in the treatment of breast cancer. Early detection of lymphedema during routine examinations will be useful for treatment management and prevention of complications. In our study, it was found that lymphedema had a negative effect on quality of life by affecting shoulder, arm, and hand functions, even in the early stages. Early diagnosis and raising awareness of well-known risk factors for lymphedema should be new targets of treatment.

## Figures and Tables

**Table 1:**
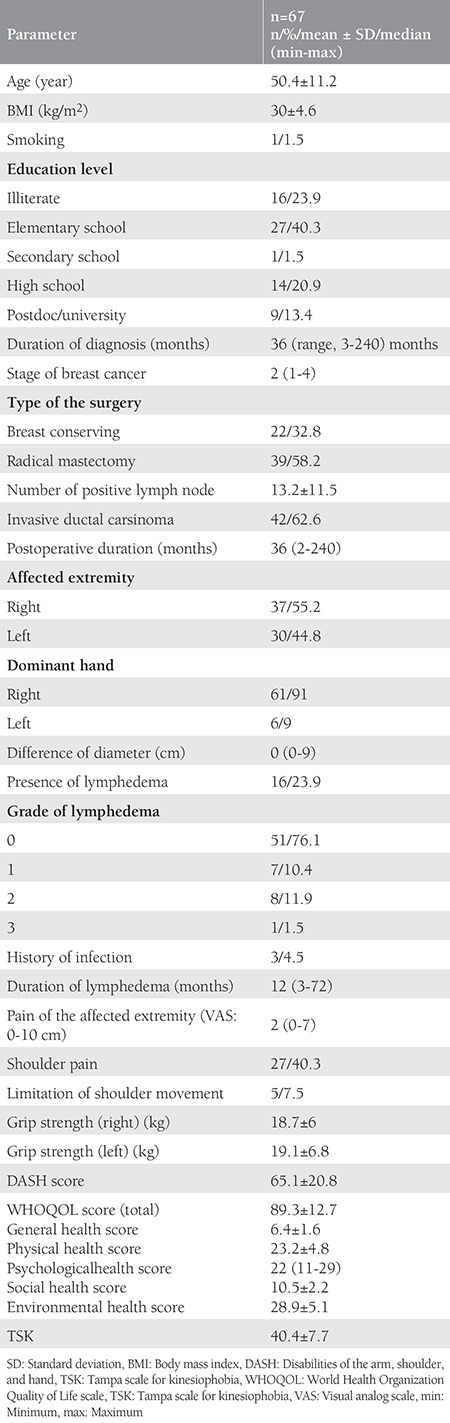
Descriptive characteristics of the study group

**Table 2:**
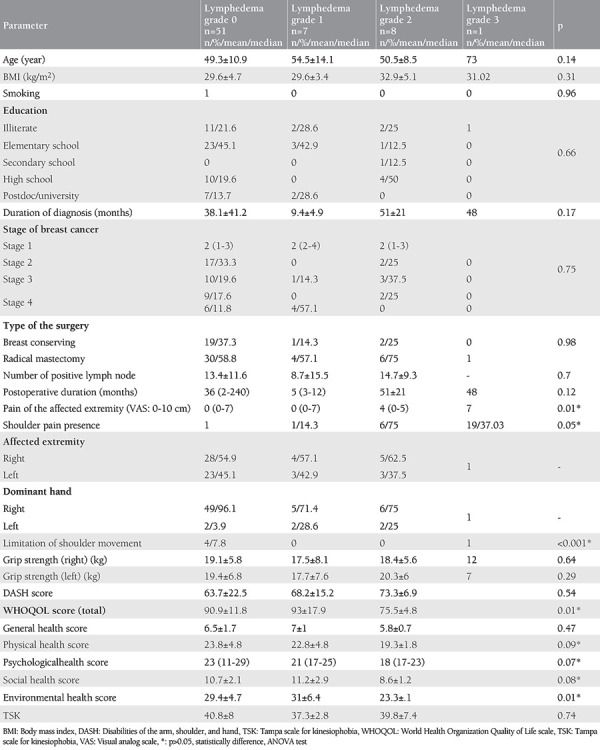
Analytic characteristics of the groups accroding to grade of the lymphedema

**Table 3:**
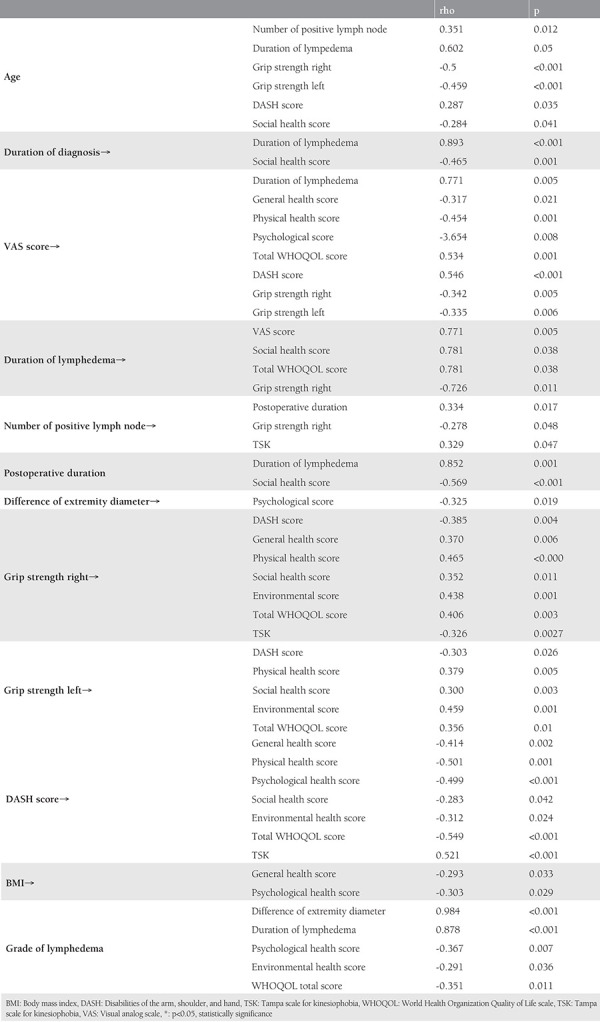
Correlation analysis of the parameters (*only statistically significant results)
